# Climate-smart precision poultry farming: artificial intelligence and sensor-based microclimate control for enhancing welfare and production efficiency

**DOI:** 10.3389/frai.2026.1972681

**Published:** 2026-10-27

**Authors:** Kelvin Ngongolo, Kasim Sakran Abass

**Affiliations:** 1 Department of Biology, College of Natural and Mathematical Sciences, University of Dodoma, Dodoma, Tanzania; 2 Department of Physiology, Biochemistry, and Pharmacology, College of Veterinary Medicine, University of Kirkuk, Kirkuk, Iraq

**Keywords:** bio-acoustic monitoring, edge computing, multi-modal data fusion, smart ventilation systems, thermal stability

## Abstract

**Introduction:**

Climate-aware farming has proved to be an innovative way of improving animal welfare and productivity on intensive broiler farms. Innovations in the Internet of Things (IoT) and artificial intelligence (AI) can provide smart systems for monitoring and predicting microclimates.

**Methods:**

A systematic review of IoT- and AI-enabled microclimate regulation for broilers was conducted according to the PRISMA-ScR guidelines. The literature reviewed was assessed based on its sensing, networking, and data processing capabilities.

**Results:**

The reviewed solutions combined IoT and AI technologies with environmental sensors, using the LoRaWAN, ZigBee, and NB-IoT communication protocols. Data analysis employed methods such as LSTM, YOLO, and fuzzy logic. Technology evolution is evident in the transition from threshold to predictive climate management, which has contributed to increased thermal stability within ±0.5 °C, more even bird distribution, reduced stress-related behaviours, and improved feed conversion ratios of 1.42–1.50 and daily weight gains of 64–68 g/day.

**Discussion:**

Although IoT and AI technologies could greatly improve broiler welfare and production effectiveness, there are still obstacles such as sensor ageing, connectivity issues in rural areas, and expensive implementation.

## 1 Introduction

The international poultry industry is one of the fastest-growing sectors within animal husbandry. It is an essential component of global food security, providing a cost-effective source of animal protein (Wongtangtintharn et al., 2025). In order to cope with the rapid rise in demand, commercial farms have become increasingly dependent on high-density concentrated housing facilities. Unfortunately, such facilities substantially increase vulnerability in terms of management, particularly about environmental regulation, health protection, and animal welfare. Birds possess highly sensitive thermoregulatory mechanisms, making them susceptible to changes in their immediate environment (Apalowo et al., 2024). Excessively hot or cold temperatures, high relative humidity levels and an increased presence of potentially dangerous gases, including CO_2_ and NH3 released from decomposition, cause significant physiological stress (Küçüktopçu et al., 2024). This leads to numerous system disruptions, compromised immunity and mass mortality rates, as well as decreases in production indicators such as FCR and DWG (Nasir, 2025).

The traditional method of environmental regulation on poultry farms uses threshold-based microclimate control systems that are reactive in nature. These systems employ simple thermostatically controlled mechanisms that activate fan ventilators and radiant heaters when environmental parameters exceed the preset limits (Rowe et al., 2019). These mechanisms are extremely unsuitable for modern precision agriculture practices due to their failure to consider spatial variability within the farm shed, variable environmental conditions outside and the behavioral responses of the flocks.

To bypass such limitations, the concept of precision poultry farming (PPF) is gaining traction, ushering in a crucial shift from manual, reactive farming practices to automation and data science (Paneru et al., 2026). Contemporary intelligent poultry farms are able to set up non-invasive monitoring networks by integrating the Internet of Things (IoT) alongside artificial intelligence (AI; Toth et al., 2025). Microclimatic and atmospheric parameters are measured using special sensors placed across a heterogeneous grid, while avian activity is tracked using computer vision technology and bio-acoustic telemetry systems (Fan et al., 2025; Manikandan and Neethirajan, 2025). Combined with edge computing and sophisticated data processing technologies, such as LSTM neural networks, YOLO vision algorithms and fuzzy logic controllers, the data can be translated into valuable predictive analytics (Eskioglu, 2025; Rahayu et al., 2025). This enables climate control devices to make pre-emptive and homeostatic adjustments to the microclimate, thereby minimizing energy consumption and enhancing thermal comfort.

Although the technological advantages of the IoT architecture based on artificial intelligence are evident, its rapid adoption is facing social and technological challenges (Mata, 2026). Challenges related to the rapid deterioration of sensors in challenging ammonia-rich environments, limited connectivity in remote rural areas, power grid risks, and substantial investment in IoT technology development still hinder its market expansion (Taer and Taer, 2025; Mushtaq et al., 2025). To help bridge these gaps, this systematic scoping review outlines the interaction between modern computer science and advanced intensive farming practices. Following the PRISMA scoping review approach, this paper critically analyses studies on sensor network design and machine learning algorithm application from 2023 to 2026. The objectives of this review are to: (1) evaluate and categorize multi-level IoT and AI processing structures, (2) estimate the data-driven effects of microclimate optimization on biological results, and (3) outline current limitations to provide a basis for further research into developing climate-smart poultry production facilities.

### 1.1 Problem statement

The intensive poultry industry relies on outdated microclimatic designs that depend on threshold-based, reactive thermostats. Traditional models are incapable of addressing spatial diversity, changing weather conditions, and the accumulation of toxic gases such as ammonia in the atmosphere. This can lead to severe thermal stress in animals, resulting in unusual behaviors, distressing vocalizations, immune deficiencies, increased mortality rates, and inefficient feed consumption. Manual management techniques are unable to provide timely solutions, leading to heavy financial losses and poor animal welfare conditions.

### 1.2 Significance of the study

This study aims to address important gaps at the intersection of engineering and agriculture by designing an IoT and AI framework for smart poultry housing. By synthesizing empirical knowledge on predictive and homeostatic control for microclimatic optimization, this review establishes benchmarks for the efficient use of feed conversion rate (FCR) and daily weight gain (DWG). Additionally, it provides a technical and economic strategy to counteract issues such as sensor degradation and the prohibitive cost of implementation.

### 1.3 PICOS research question

This scoping review aims to answer the following research question: What is the effect of applying AI and IoT-enabled technologies for microclimate monitoring and control, including sensor networks, machine learning algorithms, computer vision technology and intelligent ventilation control, compared with conventional threshold- or manual-based environmental control systems, in terms of improved welfare, environmental control and production efficiency? Outcome measures of interest include thermal regulation, air quality control and indicators of welfare in terms of bird behavior and vocalizations in response to distress. Other measures include feed conversion ratio (FCR), daily weight gain (DWG), mortality reduction, energy efficiency and overall system performance. Technical and economic outcomes, including sensor durability connectivity efficiency, power consumption, implementation cost, and ROI will also be considered. Eligible information sources consist of peer-reviewed experimental studies, field trials, observational studies, engineering evaluations, architectural evaluations, and conference proceedings conducted between 2015 and 2026.

### 1.4 Contributions to climate-smart precision agriculture

This research represents a significant step forward in developing climate-smart precision farming by providing an IoT- and AI-powered, hierarchical microclimate control system for intensive poultry farming. Unlike reactive systems based on thresholds, the authors present a predictive architecture utilizing LoRaWAN, ZigBee, and NB-IoT communication protocols, as well as state-of-the-art machine learning algorithms such as LSTM, YOLO and fuzzy logic.

The main contribution lies in demonstrating how multimodal data fusion can ensure a temperature range of ±0.5 °C, thereby preventing heat stress in birds and significantly improving their wellbeing. This engineering advancement brings clear bio-economic advantages, including improved feed conversion rates of 1.42–1.50 and weight gain rates of 64–68 g/day.

Furthermore, the paper addresses critical challenges such as sensor decay, rural network limitations, and power shortages, offering an action plan incorporating edge computing, solid-state NDIR sensors, and solar microgrids. Relating to SDGs 2, 9 and 13, this research provides a scalable solution for sustainable livestock farming in adverse conditions.

## 2 Materials and methods

### 2.1 Study design

This systematic scoping review aims to analyze, categorize, and appraise emerging and complex literature at the intersection of artificial intelligence (AI), Internet of Things (IoT) architectural design, and precision poultry farm management. Given that smart agricultural engineering involves multiple areas, ranging from wireless sensor telemetry and computer vision to avian physiology and production economics, a systematic scoping review was deemed more appropriate than a meta-analysis. This approach enables the combination of highly heterogeneous sources of information, including qualitative studies, algorithmic frameworks, and quantitative production metrics. The review's structure adheres to the PRISMA extension for scoping reviews. In accordance with these guidelines, the review was conducted through a systematic mapping process involving three key steps: (1) creating a search matrix encompassing perception, network and processing layer architectures; (2) applying stringent criteria to identify high-quality empirical research papers; and (3) combining technical issues with physiological and economic results to develop a strategy for future research.

### 2.2 Keywords and search strategy

To ensure a rigorous, systematic, and reproducible literature search, a systematic search matrix was developed using Boolean operators from primary databases, namely Google Scholar, Scopus, Web of Science, and IEEE Xplore. The systematic search approach was divided into specific conceptual sections to effectively identify relevant articles. In the first block, the area of facilities was outlined using the string (“poultry” OR “broiler” OR “chicken house” OR “poultry housing” OR “barn” OR “broiler house”). The second block concentrated on identifying infrastructure technologies, which included technology infrastructure, hardware, and networks, using the following search terms (“Artificial Intelligence” OR “AI” OR “Internet of Things” OR “IoT” OR “sensor array” OR “edge computing” OR “machine learning” OR “computer vision” OR “wireless network”).

These blocks were merged into a single consolidated string: (“poultry” OR “broiler” OR “chicken house”) AND (“Artificial Intelligence” OR “AI” OR “Internet of Things” OR “IoT” OR “sensor array” OR “edge computing”) AND (“microclimate” OR “ventilation” OR “climate control” OR “ammonia” OR “thermal stress”). The timeframe was limited to works up to early 2026, giving preference to peer-reviewed scientific articles, book chapters, and high-impact conference papers. The database search was further complemented by backward and forward citation tracking of key review papers.

### 2.3 Inclusion and exclusion criteria

To define scientific validity for the selection criteria, strict inclusion boundaries were set. Studies were selected based on their specific system design, type of animal rearing, and availability of verifiable measurements.

*The inclusion criteria were as follows*

Papers were considered eligible if they assessed multi-sensor networks, wireless communication (e.g., LoRaWAN and Zigbee) or machine learning algorithms operating under the conditions of commercial broiler houses. The selected studies were required to provide verifiable performance metrics, such as production efficiency (FCR, DWG), bird welfare, technical limitations, and return on investment. All articles had to be peer-reviewed and published in scientific journals, books or conference proceedings that were published between 2015 and 2026 to remain relevant.

*Exclusion criteria*

Studies that did not focus on commercial poultry farms, but rather on backyard and open-range systems, were excluded. Papers studying agricultural microclimates lacking automation for poultry houses (e.g., greenhouses and ruminant barns) were also excluded. Additionally, any unreviewed or non-peer-reviewed paper that was not published in a scientific source or written in English was excluded.

*Selection justification*

In line with the standard PRISMA extension methodology for scoping reviews (PRISMA-ScR), no formal tool was used to assess risk of bias or methodological quality. Instead, the validity and quality were controlled by applying strict inclusion criteria, ensuring that only peer-reviewed articles, confirmed conference papers, and reputable book chapters from 2023 to 2026 were included.

### 2.4 Data synthesis

A complex thematic synthesis strategy was adopted to systematically analyze how the extracted literature related to the primary research objectives. Firstly, the various hardware and architectural components were organized into a three-level taxonomy: environmental sensor data was grouped into the perception layer, telemetry configurations into the network layer, and algorithm models into the processing layer. Secondly, real-world data were gathered to quantify the biological effects of the downstream analysis by comparing traditional threshold-based microclimates with AI-designed homeostatic conditions in terms of feed conversion ratio (FCR), daily weight gain (DWG), and welfare outcomes for poultry. Thirdly, technical barriers such as sensor degradation, connectivity restrictions in rural areas, potential power supply infrastructure vulnerabilities, and cost implications were identified. Although the review process was not registered in a public repository prior to the start of the study, the scoping review was conducted in strict accordance with the PRISMA Extension for Scoping Reviews (PRISMA-ScR) to guarantee full methodological transparency and replicability.

## 3 Results

In line with the PRISMA-ScR guidelines, we used a flow diagram to follow a consistent literature identification process (Figure 1). The first step involved identifying 385 studies via targeted database searches (including PubMed, Scopus, IEEE Xplore, and Google Scholar, as well as gray literature). After eliminating duplicates, 182 records (47.3%) were deleted, along with a further 56 (14.5%) due to incomplete metadata or irrelevance. Consequently, a total of 147 records (38.2%) were retained for title/abstract screening.

**Figure 1:**
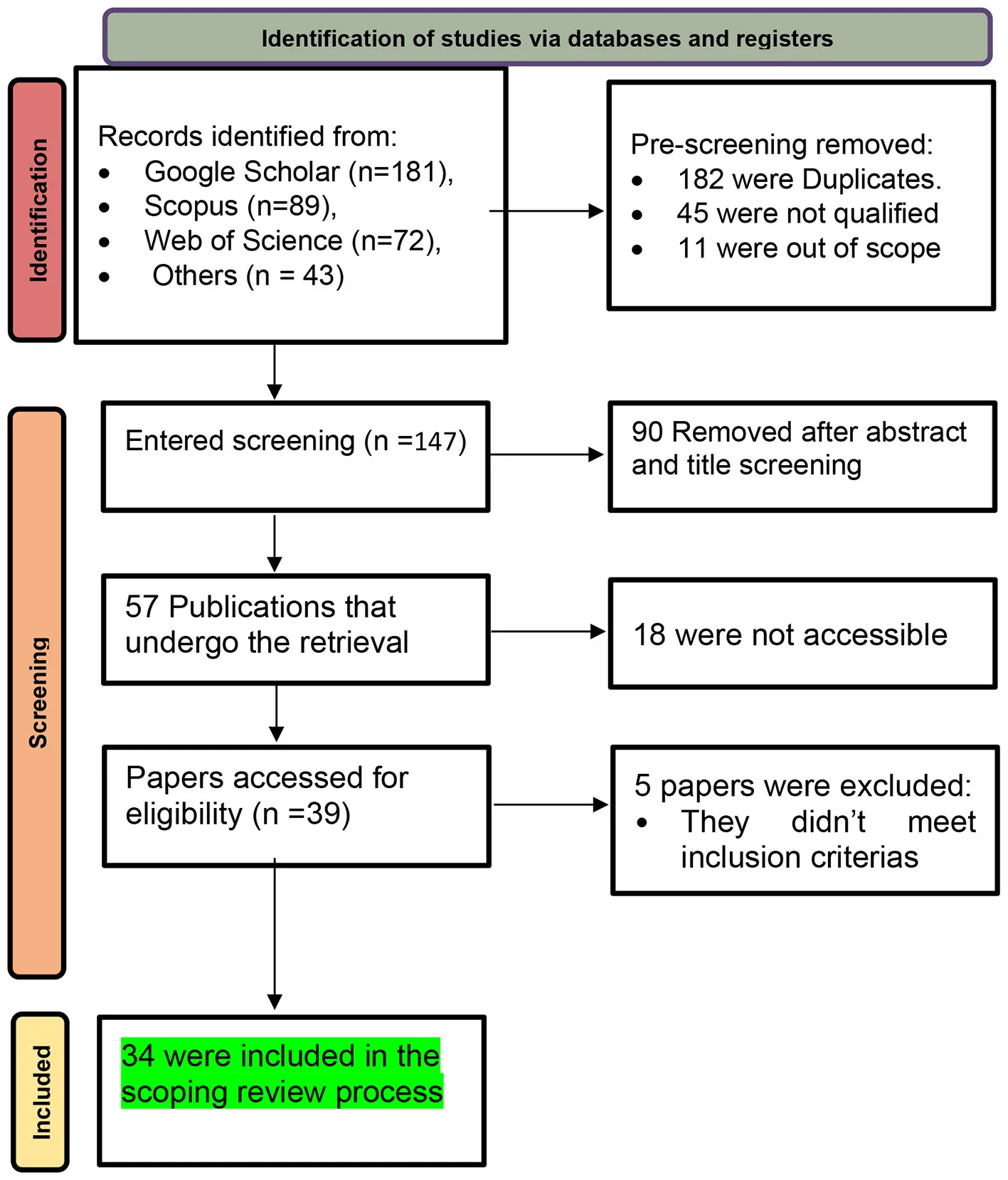
PRISMA-ScR flow diagram detailing the systematic literature identification, screening, exclusion, and final inclusion process for the scoping review.

Of these, 90 studies (61.22% of those screened) were removed due to their low relevance to smart farming, power infrastructure, or economic modeling purposes. Consequently, only 39 studies (26.53%) progressed to full-text screening. After assessing the 39 selected papers, five publications (18.24%) were eliminated as they did not sufficiently match our topic. Ultimately, 34 papers (5.7%) were included in our scoping review (Figure 1).

### 3.1 Evaluation of IoT and AI architectural frameworks in precision poultry housing

The modern intelligent chicken house incorporates an integrated IoT-AI platform that takes environmental control beyond the basic threshold-based system to a more advanced multi-attribute prediction model. As shown in Figure 2 below, the platform operates through different layers of functionality. The first layer is the perception or sensing layer. This works using the heterogeneous space grid outlined in Table 1 to collect real-time microclimatic, atmospheric, visual and bio-acoustic data.

**Figure 2:**
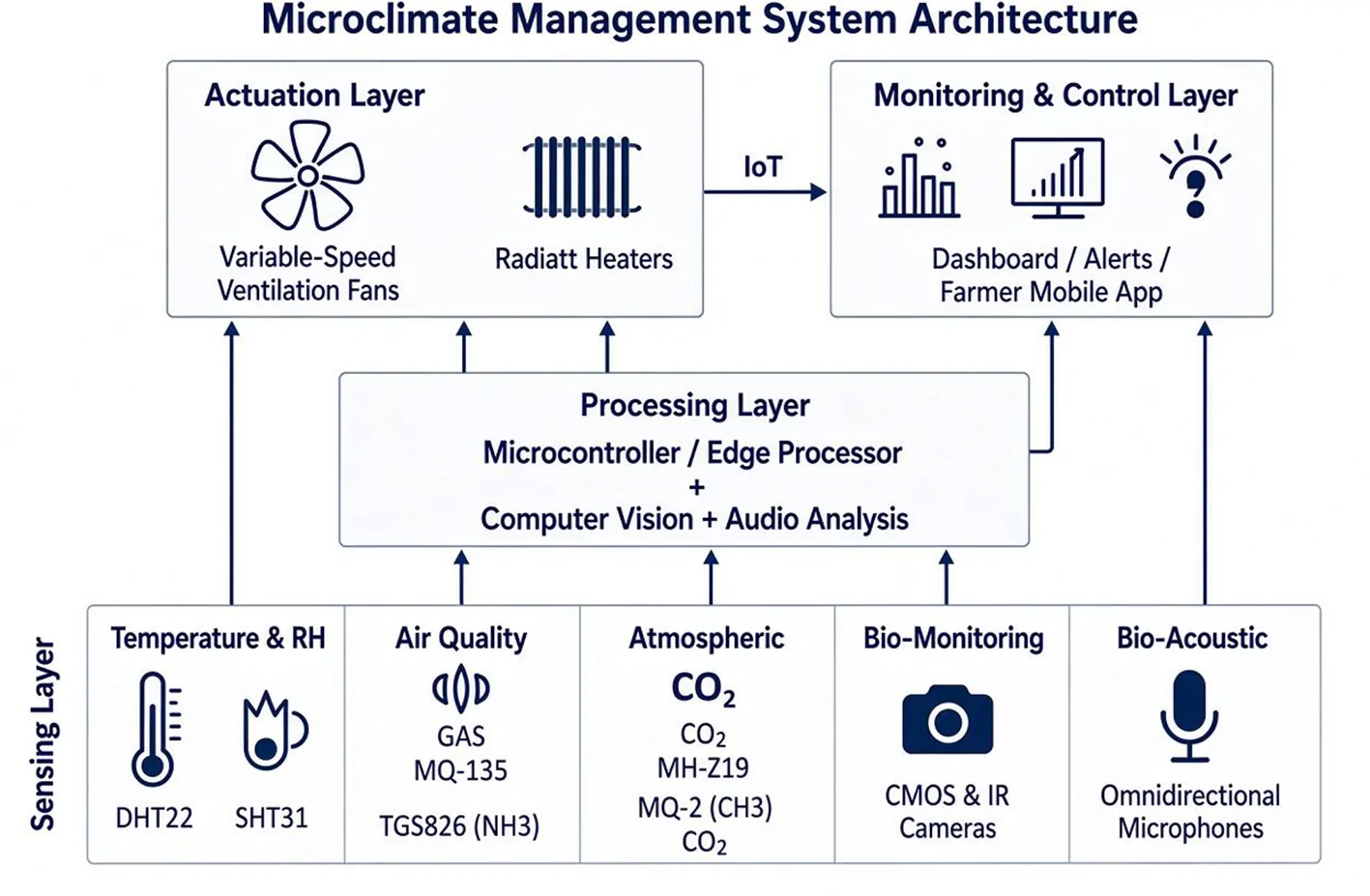
The architecture of an intelligent microclimate control system for poultry shows the hierarchical nature of integration between the sensing, processing, actuation, and monitoring layers. This figure represents the hierarchical architecture of the microclimate control system. The sensing layer is responsible for collecting environmental data, gases and audio-visual welfare indicators. This data is then processed by an edge processor at the processing layer using computer vision and audio analysis. Real-time decisions are sent from the processing layer to the actuation layer for climate regulation. At the same time, the monitoring and control layer is updated with telemetry.

**Table 1:** Taxonomy of environmental and bio-monitoring sensor arrays in precision poultry housing: specifications, operational automation functions, and supporting literature.

Category	Sensor type	Target parameters	Function in automation	References
Microclimate	DHT22, SHT31, RTD	Temperature & relative humidity	It regulates environmental parameters via the dynamic control of variable-speed ventilation fans and radiant heaters, achieving optimal thermal comfort	Küçüktopçu et al., 2024; Paneru et al., 2026; Tedeschi et al., 2025
Air quality	MQ-135, TGS826	Ammonia (NH_3_)	It enables high-volume air exchange to prevent excessive ammonia levels caused by litter decomposition, which can lead to health issues	Abdel-Wareth and Ahmed, 2026; Küçüktopçu et al., 2024; Ni, 2024
Atmospheric	MH-Z19, MQ-2	Carbon dioxide (CO_2_) & methane (CH_4_)	It calculates the minimum ventilation required to ensure sufficient oxygen is present without causing thermal stress due to drafts	Eskioglu, 2025; Küçüktopçu et al., 2024; Ni, 2024
Bio-visual monitoring	CMOS, infrared (IR) cameras	Avian distribution & activity	It can work in combination with computer vision software to detect behavioral abnormalities (e.g., crowding caused by cold or wing flapping due to heat)	Fan et al., 2025; Paneru et al., 2026; Tedeschi et al., 2025
Bio-acoustic	Omnidirectional condenser microphones	Stress vocalizations and distress calls	It recognizes abnormal noise (e.g., distress calls or coughing), allowing immediate action via control system overrides	Eskioglu, 2025; Manikandan and Neethirajan, 2025; Paneru et al., 2026

Network layer coordination is required for network backhaul. As the comparative data in Table 2 illustrates, the choice of communication protocol depends on farm topography: LoRaWAN is used in large-scale facilities with structural insulation; ZigBee is used for high-density indoor sensor mapping; and NB-IoT is used to simplify cloud integration for remote management systems. Finally, all these data streams converge at the processing layer, as shown in Table 3. Here, edge processors and various machine learning models, including LSTM forecasting, YOLO computer vision, and fuzzy logic inference, process real-time risk and distress indicators of the fowls.

**Table 2:** Comparative analysis of wireless communication protocols in precision poultry farming: network topologies, technical advantages, deployment contexts, and empirical literature.

Protocol	Topology type	Technical advantage	Primary deployment context	References
LoRaWAN	Star-of-stars	The SubGHz radio frequency range provides extended distance and better penetration capabilities through the thick materials used in barns, such as concrete and metallic insulation	The multiple poultry houses are interconnected within large, geographically dispersed commercial farming establishments	Abdel-Wareth and Ahmed, 2026; Condotta and Tedeschi, 2026; Paneru et al., 2026
ZigBee	Mesh network	It is a power-efficient network with a self-recovery mesh topology that supports dynamic multi-hopping and data routing across dispersed sensor nodes	There is a dense deployment of sensors inside for fine environmental monitoring, such as temperature and humidity mapping	Abdel-Wareth and Ahmed, 2026; Fan et al., 2025; Paneru et al., 2026
Cellular IoT (NB-IoT)	Point-to-point cellular	It establishes direct long-distance connectivity to cloud services, eliminating the need for a gateway node	Integration with a centralized, cloud-based database management system is essential for off-site or contracted farms	Condotta and Tedeschi, 2026; Eskioglu, 2025; Ni, 2024

**Table 3:** Taxonomy of artificial intelligence and machine learning architectures for precision poultry automation: mathematical frameworks, operational goals, control logic, and supporting literature.

Model category	Core mathematical architecture	Operational goal	Automation logic	References
Time-series forecasting	LSTM/GRU networks	Prediction of environmental parameters	It forecasts short-term variations in temperature, humidity, and gas levels (e.g., a 2-h prediction) based on past sensor values and external weather parameters	Eskioglu, 2025; Küçüktopçu et al., 2024; Rahayu et al., 2025
Deep computer vision	CNN/YOLO architectures	Extraction of real-time image features	It enables non-intrusive monitoring of flock behavior by assessing spatial distribution, clustering and posture-based thermal comfort status	Eskioglu, 2025; Fan et al., 2025; Paneru et al., 2026
Expert systems	Fuzzy logic inference	Multi-objective adaptive control	It resolves conflicting environmental control tasks (e.g., combatting heat stress vs. cold stress) via rule-based reasoning for decision-making purposes	Abdel-Wareth and Ahmed, 2026; Küçüktopçu et al., 2024; Rahayu et al., 2025
Ensemble	Random Forest/SVM	Classification of risk state	It classifies the environmental state as one of three discrete states: safe, at-risk or critical, using data from multiple sensors	Eskioglu, 2025; Küçüktopçu et al., 2024; Tedeschi et al., 2025

### 3.2 Quantifying production and welfare outcomes via intelligent microclimate optimization

Indeed, the introduction of a fully automated, integrated system that employs AI principles allows us to transform commercial broiler rearing. This is achieved by shifting from a purely reactive system based on threshold values to a predictive, homeostatic environment. As shown in Table 4, standard systems subject flocks to significant microclimate changes. This leads to piling behavior, energy wastage, and the generation of distress vocalizations due to poor air quality. In contrast, precision control systems provide thermal stability of no more than ±0.5 °C, thereby maintaining animal homeostasis and preventing the activation of heat shock proteins at a cellular level. These benefits translate into improved welfare and performance, allowing feed efficiency to be enhanced. Multimodal data integration and real-time predictive analysis of data from environmental sensors, computer vision, and bioacoustics make it possible to adjust conditions to avoid thermoregulation-related responses such as panting and respiratory alkalosis (see Figure 3). The optimization algorithm therefore provides for greater energy investment in production and less in survival mechanisms. This results in increased feed efficiency, with the FCR reaching 1.42–1.50 and the DWG rising to 64–68 g/day.

**Table 4:** Empirical comparison of production performance and avian welfare outcomes: legacy threshold systems vs. AI-automated smart climate optimization.

Core outcome component	Legacy threshold control metrics	AI-automated smart system metrics	Primary physiological/systemic mechanism	Peer-reviewed references
Thermal stabilization	There are variations in microclimate ranging from ±2.0 to 4.0 °C.	Environment with precise control and deviations of ≤ ±0.5 °C.	It decreases thermal stress and controls the overexpression of excessive heat shock proteins (HSP).	Apalowo et al., 2024; Rowe et al., 2019; Brugaletta et al., 2022
Flock distribution	There is consistent pile formation in cold areas and void formation in hot spots.	Even distribution across the usable area of ≥90%.	It eliminates crowding, which reduces stress and prevents injury from microclimates.	Li et al., 2024; Balthazar et al., 2025; Bhuiyan and Wree, 2023
Distress vocalization (acoustic indicators)	There is consistently distressed vocalizing with an increased frequency of about 4.0 kHz.	Sound monitoring with controlled ventilation and environmental management.	It detects early signs of distress and allows for the timely removal of toxic gases, such as ammonia (NH3) and carbon dioxide (CO_2_).	Rideout and Whetton, 2026; Goyal et al., 2023; Islam and Khan, 2025
Feed conversion ratio (FCR)	There is a high FCR (1.75–1.90), which reflects poor feed efficiency.	Optimized FCR is 1.42- 1.50.	It receives more calories without wasting energy on thermal regulation (e.g., panting).	Nasir, 2025; Ogbuewu et al., 2023; Wongtangtintharn et al., 2025
Production speed (DWG: daily weight gain)	A poor growth rate of 5.2–55 g/day.	Better growth performance of 64- 68 g/day on average.	Better control over the environment ensures maximum partitioning of nutrients for muscle growth.	Elwakeel, 2025; Olejnik et al., 2022; Papakonstantinou et al., 2024

**Figure 3:**
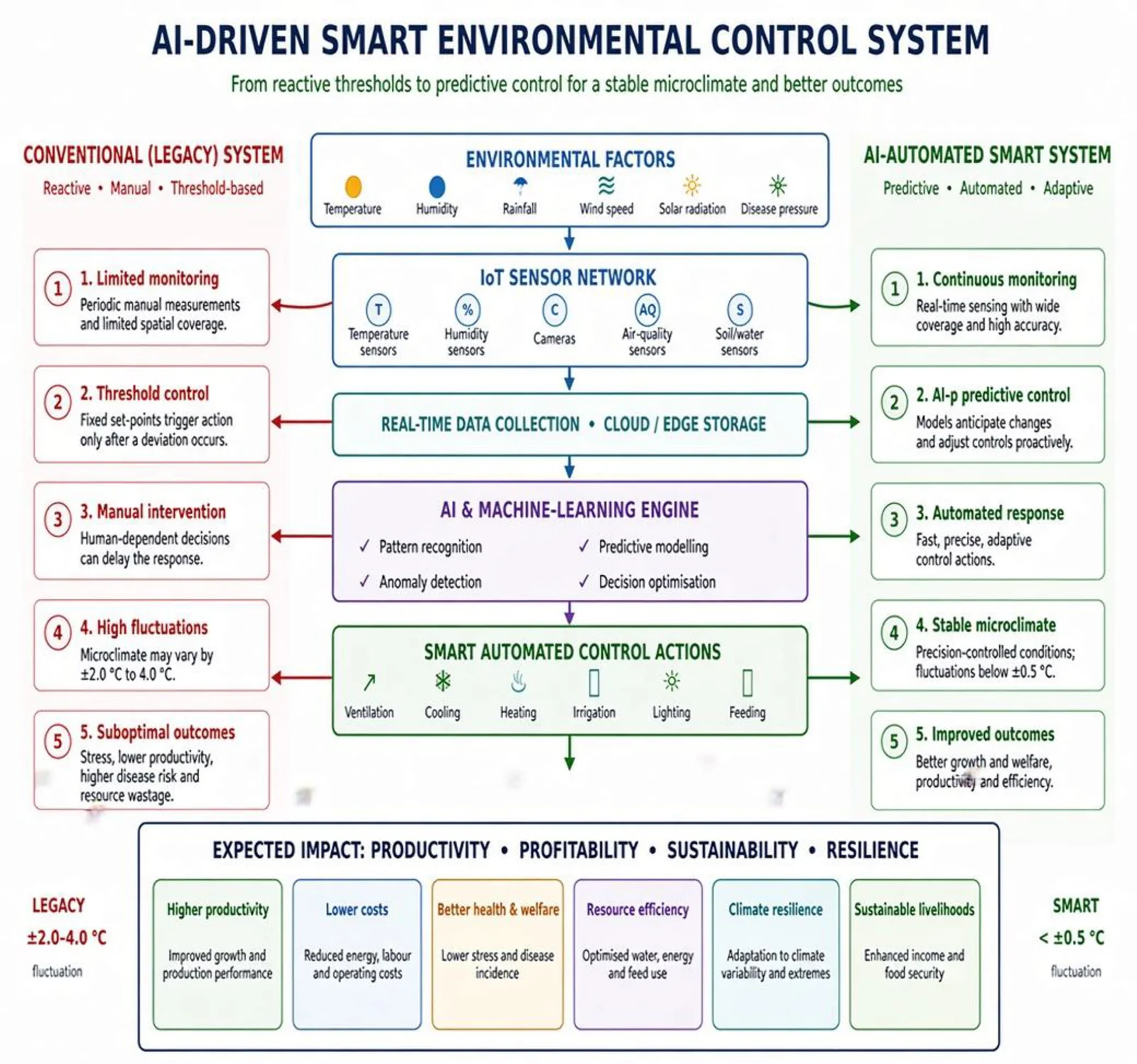
Conceptual model of an intelligent, AI-controlled environmental management system for precision microclimate control and sustainable agriculture. This figure provides a conceptual representation of how AI, IoT sensors and intelligent control systems can be used to control the environment effectively. Using AI to collect information about current environmental parameters such as temperature, humidity, and air quality enables predictions based on the results, leading to the automatic regulation of ventilation, cooling, heating, irrigation, and lighting, among other things.

### 3.3 Engineering and translational obstacles in smart poultry barns

The practical deployment of smart climate control solutions is hindered by substantial socio-economic and technical obstacles. According to Table 5, extreme conditions in poultry barns quickly result in sensor malfunction. Rural connections are unreliable, which slows down real-time computer vision processing. Heat spikes can also lead to delayed ventilation due to poor connectivity. Furthermore, inadequate electricity infrastructure on farms may result in blackouts during critical ventilation processes. This challenge can be mitigated through the installation of decentralized backup systems and microgrids for renewable solar energy, as illustrated in Figure 4.

**Table 5:** Critical evaluation of technical bottlenecks, economic trade-offs, and strategic engineering roadmaps for scalable AI-sensor integration in modern poultry facilities.

Technical & economic dimension	Current commercial implementation gap	Immediate operational impact	Short-term mitigation (1–3 years)	Long-term future roadmap (4–7 years)	References
Sensor lifespan & durability	Electrochemical and optical sensors experience rapid fouling and calibration issues after 3–6 months due to corrosive gases (NH3), moisture and the deposition of biological dust.	Increased maintenance costs; weakening of the signal resulting in either false alarms or an incorrect ventilation response.	•Hydrophobic and IP67-rated modules. •Purge mechanisms using compressed air.	•A shift toward solid-state technology and non-dispersive infrared (NDIR) sensors. •Self-calibration and machine-learning correction algorithms incorporated.	Taer and Taer, 2025; Paneru et al., 2026; Toth et al., 2025
Connectivity & bandwidth limits	Insufficient availability of high-speed communication networks in rural areas leads to unreliable data communication via Wi-Fi or cellular networks.	Lagging video analytics technology and environmental response cause poor efficacy during sudden heat or gas surges.	•LoRaWAN-based low-power wide-area network telemetry system. •Resilient on-site data buffer systems.	•Utilize edge computing capabilities at the site level. •Switch from synchronous cloud processing to asynchronous, bandwidth-limited reporting.	Shi and Dustdar, 2016; Wolfert et al., 2017; Toth et al., 2025
Power infrastructure requirements	The need for a continuous power supply for sensors and edge computing equipment poses a challenge due to inadequate rural grid infrastructure.	Greater chance of outages; ventilation will not work when needed during power outages, which harms the wellbeing of animals.	•Ultra-low power microcontrollers with energy-saving hardware. •Decentralized battery backups at node level.	•Connect to renewable, farm-based solar microgrids and battery energy storage systems. •Refine algorithms to optimize “Green AI” processing state performance.	Tang, 2015; Wang and Zhu, 2017; Mushtaq et al., 2025
Economic trade-offs and return on investment (ROI)	The high initial cost of the technology is prohibitive for small-scale producers.	Slow adoption rate and unequal distribution of precision farming solutions among different supply chains.	•Modular subscription models for hardware. •Software upgrades for existing infrastructure.	•Create standard open communication protocols. •Utilize carbon credits based on proven energy efficiencies.	Gilbert et al., 2023; Mata, 2026; Paneru et al., 2026

**Figure 4:**
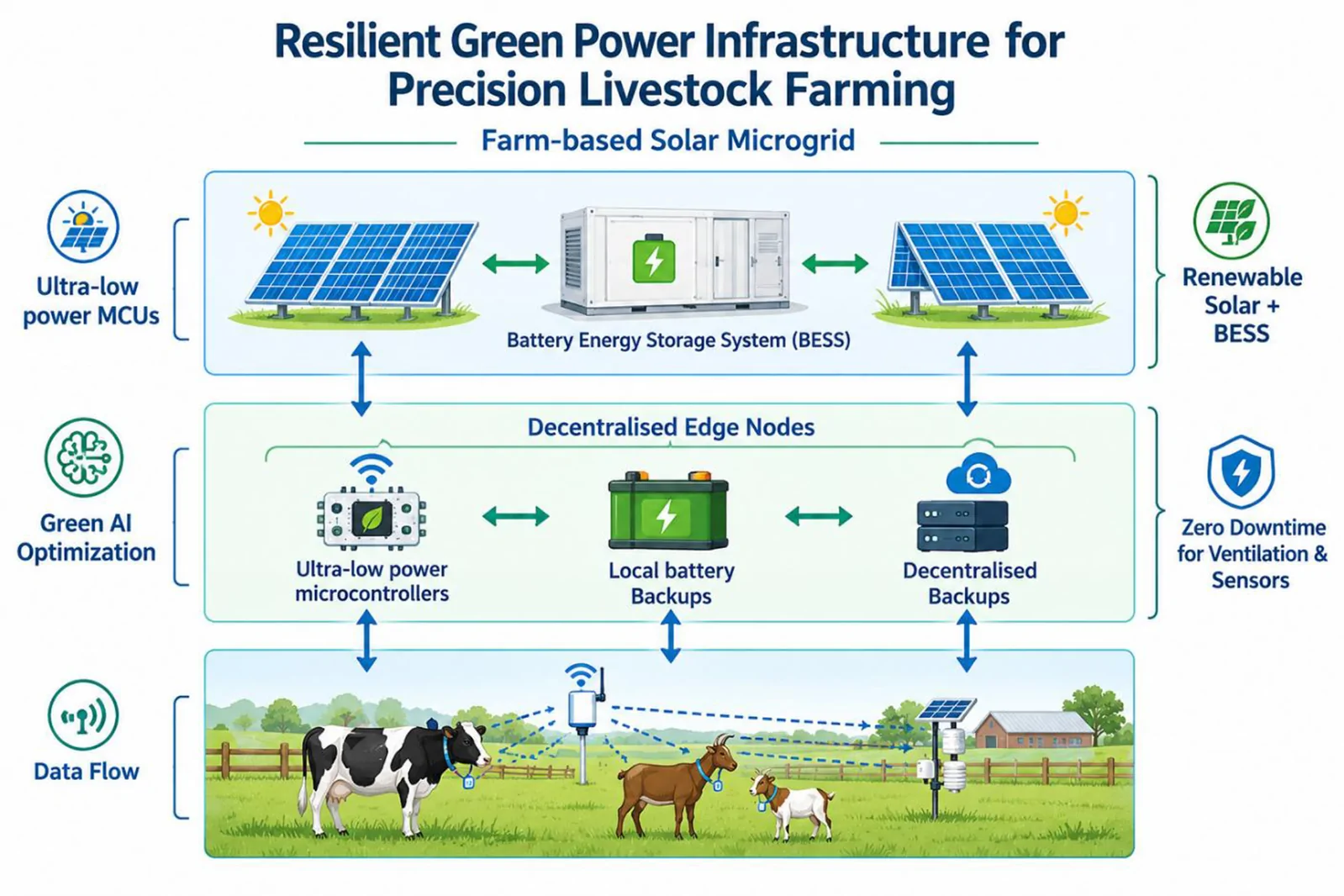
Resilient green power infrastructure for precision livestock farming.

From an economic standpoint, the cost of expensive hardware exceeds $15,000 per unit, which poses a significant challenge for smaller farms. However, this issue can be mitigated by using modular, flexible, plug-and-play models with subscription services and legacy hardware adaptation software (Figures 5, 6). The utilization of interim solutions, such as LoRaWAN and IP67 housing, increases robustness. Finally, the engineering roadmap for a long-term solution requires a transition to solid-state NDIR sensors, edge computing clusters, and standardized open communication protocols for precise poultry automation.

**Figure 5:**
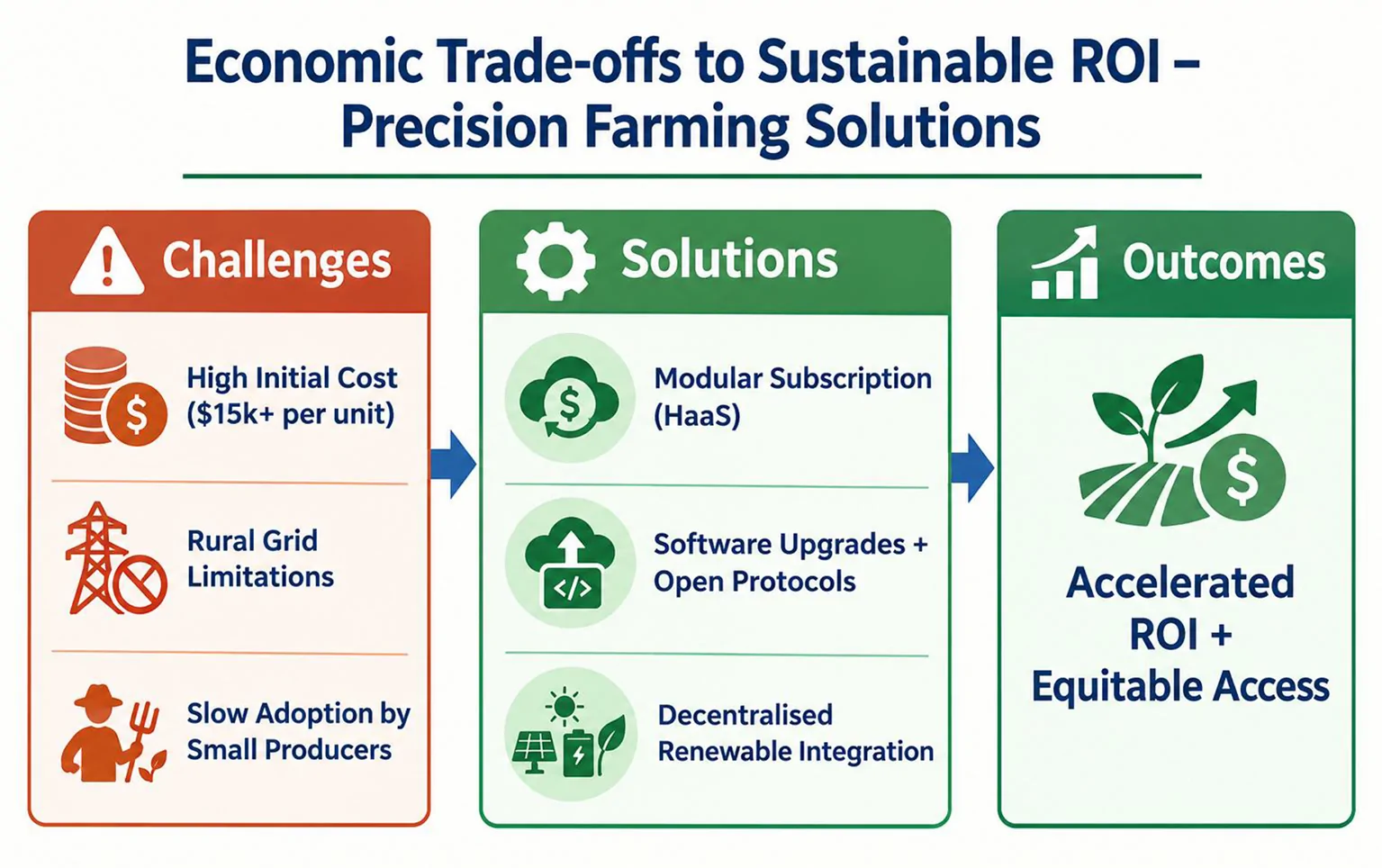
From economic challenges to sustainable ROI: precision farming solutions.

**Figure 6:**
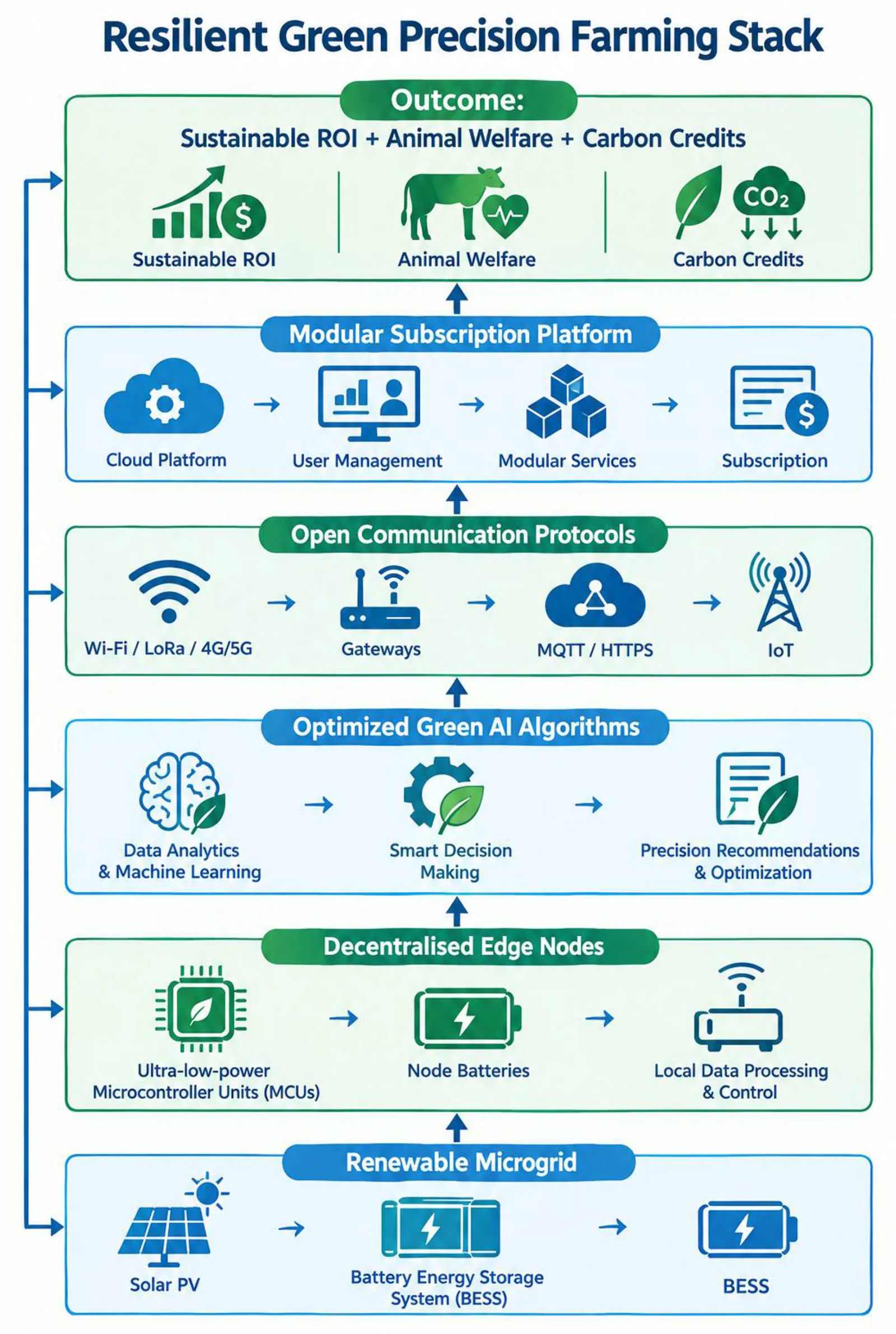
Overcoming cost barriers: modular solutions for precision livestock ROI.

## 4 Discussion

### 4.1 Multi-layered IoT–AI architecture for predictive and adaptive poultry house management

Assessing the multi-layered IoT and AI system architectures employed in contemporary intelligent poultry houses has revealed a significant paradigm shift from threshold-dependent microclimate regulation to proactive, multi-parameter predictive modeling. Using heterogeneous sensor arrays that include microclimatic, atmospheric, visual, and bio-acoustic parameters enables the development of highly adaptive systems that directly facilitate the birds' homeostasis and general welfare.

At the networking level, the targeted implementation of specific communication protocols that are suitable for the farm's topology enables the resolution of telemetry issues in agriculture. Combining LoRaWAN for long-distance transmission, capable of penetrating farm infrastructure; ZigBee for dense mesh networking inside farm buildings; and NB-IoT for cloud connection is considered the optimal approach. This approach is supported by the results of Toth et al. (2025), who demonstrated that multi-protocol architectures minimize the negative effects of signal attenuation in farm environments with metallic insulation and reinforced concrete structures. Furthermore, Bordignon et al. (2025) emphasized the importance of robust wireless topologies for achieving low-latency data exchange in agriculture.

The processing component showcases the revolutionary capabilities of integrating multi-modal AI technology in intelligent poultry farming. The combination of long short-term memory (LSTM) algorithms for time series analysis and deep computer vision methods such as YOLO and bio-acoustic recognition results in an integrated, context-based understanding of flock behavior. This supports Paneru et al.'s (2026) point that multi-modal information fusion enables a more accurate welfare assessment than sensors alone. Vision-based recognition of abnormal activities, such as crowding, together with the recognition of stress sounds using bio-acoustic analysis (Eskioglu, 2025), enables the prompt implementation of measures, offering a superior alternative to threshold-based methods. Fuzzy logic also helps to solve conflicts between multiple environmental goals, including maximizing air circulation to reduce ammonia without creating strong drafts. Studies by Küçüktopçu et al. (2024) and Rahayu et al. (2025) show that fuzzy logic can transform qualitative data into control actions, improving regulation, performance, efficiency and animal welfare.

### 4.2 Comparative performance, limitations, and operational boundaries of deployed AI models

Although LSTM and YOLO neural networks and fuzzy logic controllers are examples of advanced automation techniques, they are each characterized by unique operational limitations that govern their practical usage on commercial poultry farms. LSTMs have a high capacity to model non-linear thermal inertia and short-term microclimates (with time horizons of 1–2 h) using sensor stream data (Eskioglu, 2025; Rahayu et al., 2025). However, the accuracy of their predictions tends to decrease for longer time horizons due to error propagation and dependency on a large volume of training data. For this reason, they should only be implemented in well-insulated and structurally sound buildings.

However, their effectiveness is greatly reduced by the harsh conditions inside poultry houses, such as rapid lens contamination due to dust and ammonia mist, and obstructions caused by hanging equipment. Therefore, automated purging or the use of IP67 housings is necessary before YOLO can be used consistently. In conclusion, fuzzy logic control systems handle the trade-offs posed by the objectives of poultry houses—for example, the balance between minimum ventilation for ammonia evacuation and the risk of cold drafts—much better than conventional PID controllers, which cannot deal with the cross-coupling of multiple variables. Nevertheless, fuzzy logic systems are subject to “rule explosion” when there are more input parameters, so they must be combined with machine learning optimization layers, such as genetic algorithms and random forests.

### 4.3 Generalization, data demand, interpretability, and deployment challenges across climate zones

AI-based microclimate control systems can be very helpful in improving environmental management, animal welfare, and production efficiency in the livestock and poultry industry. However, successfully implementing microclimate control systems requires considering three essential aspects: the generalizability of models to various climates, the availability of sufficient training data, and the explainability of the models' decision-making processes. Models built using controlled conditions or in specific geographical areas may not perform well in other environments with different climatic features, management practices, or housing types (Tedeschi et al., 2021; Rosa, 2021).

One of the key limitations of AI-based environmental control systems is their limited ability to generalize across different climatic conditions. Models built based on data obtained in temperate areas may be ineffective in tropical zones, which are characterized by high temperatures and humidity, and where heat stress and ammonia production are common problems. Arid zones are also unique in terms of problems related to extreme temperature changes throughout the day and a lack of water. All these climatic factors affect the way animals react and how buildings perform, resulting in models built under different conditions being inaccurate. To address this, scientists are promoting techniques such as adaptive learning, transfer learning, and domain adaptation (Tedeschi et al., 2021; Menezes et al., 2024).

A further problem is the significant amount of data and computation involved in using highly advanced deep learning algorithms. Algorithms such as LSTM and YOLO require large volumes of sensor and image data for training and testing purposes. Furthermore, gathering labeled data on animal health, welfare, and behavioral issues is costly and time-consuming. These models also require substantial computational resources to operate in real time. Easier methods, such as Random Forests and fuzzy logic systems, require smaller amounts of data and computing power. However, the broader use of AI technology also requires models to be more transparent. Deep learning models are often considered “black boxes” due to the lack of clarity regarding the reasons behind particular actions. The introduction of Explainable Artificial Intelligence (XAI) technologies, such as SHAP and attention-based visualization, will facilitate the responsible deployment of AI decision-making systems and increase user trust (Tedeschi et al., 2021; Yaseer and Chen, 2021; Guo et al., 2026).

### 4.4 AI-driven climate optimization and welfare-centric performance gains in smart poultry systems

As the empirical results in Table 4 show, switching from standard threshold-based HVAC regulation to climate optimization via artificial intelligence improves the welfare and productivity of broilers. Specifically, by ensuring an optimal thermal equilibrium of ≤ ±0.5 °C, the system eliminates the significant microclimate changes (±2.0–4.0 °C) that are characteristic of traditional housing units. From a physiological perspective, the system guarantees limited heat shock protein (HSP) expression, thereby preventing stress reactions related to thermoregulation, such as panting and respiratory alkalosis. This finding is consistent with the results of recent research by Apalowo et al. (2024) and Rowe et al. (2019), who demonstrated that stable thermal conditions prevent gut damage and inflammatory processes. At the same time, the equal distribution of heat across ≥90% of the floor area reduces behaviors such as huddling, as demonstrated by the results obtained by Li et al. (2024) and Balthazar et al. (2025).

Fusing bioacoustics with environmental information adds another dimension to real-time risk management. Bioacoustic sensing enables ventilation changes to be made in real time, effectively decreasing levels of NH3 and CO_2_, as indicated by studies carried out by Islam and Khan (2025). This conserves the energy required for temperature regulation, allowing resources to be channeled into growth. Such processes can lead to optimal conversion ratios and increased body weights, as suggested by studies conducted by Nasir (2025) and Wongtangtintharn et al. (2025).

### 4.5 Implementation challenges and scalability barriers in IoT–AI-enabled poultry systems

Despite their potential benefits, the implementation of innovative IoT and AI technologies in industrial poultry farming faces several engineering and socio-economic barriers. While the importance of accurate climate control for animal comfort and productivity is beyond question, the inner setting of poultry farms creates a challenging operating environment for electronic devices. As illustrated in Table 5 below, high levels of corrosive gas (NH3) and moisture, along with biological dust, result in frequent sensor calibration failures and rapid sensor degradation within 3–6 months. Clearly, this hinders the effective operation of IoT and AI equipment.

These findings correlate with the results of previous research. According to Paneru et al. (2026) and Toth et al. (2025), conventional sensors typically malfunction or produce unreliable data when exposed to high ammonia concentrations unless protected by special coverings. Immediate solutions include using hydrophobic and IP67-certified modular casings, as well as air purification techniques, to protect sensors. However, a more efficient option may be to use durable solid-state and non-dispersive infrared sensors.

Alongside hardware issues, connectivity problems in rural production areas greatly impact the timely processing of data, particularly for high-throughput applications such as computer vision. Cloud computing adds to latency, which can result in delayed control actions in response to sudden changes in environmental conditions. Shi and Dustdar (2016) and Wolfert et al. (2017) suggest that these issues can be resolved using edge computing frameworks, which allow for instantaneous control actions and reduce bandwidth requirements through selective cloud synchronization.

Economic factors continue to present challenges to the adoption of AI-based precision poultry farming. The deployment of automated sensors, computer vision technology, environmental control technologies and decision-support systems requires significant investment in equipment, software, network infrastructure and expertise. While larger companies can offset this investment through increased productivity, improved animal health and greater efficiency, smaller producers may struggle to do so (Tedeschi et al., 2021; Rosa, 2021). Furthermore, additional costs are incurred in relation to maintenance, data management, and system integration. Researchers have suggested approaches to more affordable deployment, such as hardware as a service (HaaS), retrofitting existing facilities using a modular approach, and interoperable standards (Tedeschi et al., 2021, 2025; Yaseer and Chen, 2021; Menezes et al., 2024).

### 4.6 The contextual utility of legacy threshold-based systems

Despite the many advantages of AI-based IoT systems for microclimate control, traditional threshold-controlled environmental management systems remain important in certain production environments. For example, in the case of small-scale poultry farms operating under subsistence conditions, limited funding, technical skills, and digital technology can hinder the implementation of sophisticated precision management technologies. In such cases, simple thermostatic regulators offer a cost-effective and reliable means of managing basic environmental conditions, eliminating the need for cloud-based solutions, complex sensors, and technical personnel. Moreover, stable power sources and internet access are often unavailable in rural areas, making the deployment of data-intensive AI impossible (Tedeschi et al., 2021, 2025; Rosa, 2021).

Legacy systems are also applicable to naturally ventilated poultry houses, where simpler environmental controls are needed. Mechanical thermostats and electromechanical controllers consume low levels of energy and are less susceptible to problems related to network failures, software issues, and sensor deterioration. Furthermore, in temperate climate zones with fairly consistent weather patterns, the increased productivity attained through AI-based predictive control may not offset the higher costs and greater complexity of these systems. Therefore, although AI-enabled systems represent the future of precise poultry farming, legacy systems remain applicable due to their cost-effectiveness, reliability and accessibility (Yaseer and Chen, 2021; Tedeschi et al., 2021, 2025; Menezes et al., 2024).

### 4.7 Ethical dimensions and socio-technical implications of precision livestock farming

Precision livestock farming techniques using AI offer many advantages in terms of productivity and environmental management. However, there are some major ethical and societal considerations regarding precision livestock farming. For example, the lack of appropriate governance policies could affect data ownership and privacy, given that modern IoT devices provide constant data collection on farm activities, animals and the environment, which is usually stored in the cloud. Furthermore, ethical concerns arise when considering the algorithmic bias of AI, since most AI models are trained using data from large commercial farms. Consequently, using such algorithms on small farms or farms with less standard conditions can result in erroneous recommendations. Finally, excessive attention to optimizing production processes can affect animal welfare, to the point that animals may be unable to behave normally in a strictly controlled environment. In other words, effectively implementing precision livestock technology requires an adequate data governance policy and the proper development of AI models (Tedeschi et al., 2021; Rosa, 2021; Menezes et al., 2024).

### 4.8 Limitations, future research directions, and practical recommendations

The scoping review has several limitations that should be considered when interpreting the analysis's results. Firstly, all of the studies were based solely on secondary sources of information, with no primary experimental validation of the suggested IoT and AI technologies. Consequently, it is not possible to determine any causal relationships between the application of certain technologies and the productivity of poultry farms. Secondly, the included studies exhibited methodological heterogeneity concerning system designs, sensor technologies used, machine learning techniques, environment, and performance criteria. While methodological heterogeneity is typical of research in precision poultry farming due to its interdisciplinary nature, it complicates direct comparisons between studies. Furthermore, as most applications have been carried out under pilot or experimental conditions, they cannot be generalized to industrial settings.

Further studies should focus on large-scale, longitudinal field assessments of the technical reliability, cost-effectiveness and welfare implications of AI-based environmental control systems in various climatic and managerial settings. Further research is needed to standardize methods for calibrating sensors, collecting data and communication protocols, ensuring greater compatibility between different platforms. In terms of technology, improving the durability of sensing components, such as solid-state and NDIR sensors, is crucial for use in highly toxic environments, such as poultry houses with high ammonia levels. Edge computing technology could be employed to minimize latency, enhance reliability, and reduce the reliance on rural internet connectivity. Economically speaking, modular hardware systems and a subscription service deployment model that adapts to existing poultry farm structures could benefit small- and medium-scale producers.

## 5 Conclusion

In conclusion, this study presents an innovative approach to climate-smart poultry production by introducing integrated IoT-AI systems in poultry houses. This constitutes a paradigm shift toward precision livestock farming practices. Unlike traditional threshold-based control mechanisms, the integration of multi-layer IoT-AI systems enables predictive, adaptive, and animal welfare-oriented approaches to microclimate control. Using sensors and IoT-AI algorithms such as LSTM, YOLO, and fuzzy logic makes decision-making more reliable and ensures significant stability in environmental parameters.

Empirical findings prove that IoT-AI systems positively impact animal health by reducing stress behavior, optimizing the feed conversion ratio, and increasing daily weight gain. Despite the positive aspects of using IoT-AI systems, their widespread use in livestock farming is still impeded by issues such as the lack of robust sensors, connectivity problems, and financial restrictions. To address these issues, IoT-AI requires robust sensors, edge computing technology, renewable energy sources, and more scalable models. In short, this study shows that IoT-AI offers opportunities for sustainable poultry production.

## Data Availability

The original contributions presented in the study are included in the article/supplementary material, further inquiries can be directed to the corresponding author.
